# Overview of the Mechanisms of Action of Selected Bisphenols and Perfluoroalkyl Chemicals on the Male Reproductive Axes

**DOI:** 10.3389/fgene.2021.692897

**Published:** 2021-09-27

**Authors:** Michal Ješeta, Jana Navrátilová, Kateřina Franzová, Sandra Fialková, Bartozs Kempisty, Pavel Ventruba, Jana Žáková, Igor Crha

**Affiliations:** ^1^ Department of Obstetrics and Gynecology, Faculty of Medicine Masaryk University and University Hospital Brno, Brno, Czechia; ^2^ Department of Veterinary Sciences, Czech University of Life Sciences Prague, Prague, Czechia; ^3^ RECETOX Centre, Faculty of Science, Masaryk University, Brno, Czechia; ^4^ Department of Histology and Embryology, Poznan University of Medical Sciences, Poznan, Poland; ^5^ Department of Anatomy, Poznan University of Medical Sciences, Poznan, Poland; ^6^ Department of Veterinary Medicine, Nicolaus Copernicus University, Torun, Poland; ^7^ Prestage Department of Poultry Science, College of Agriculture and Life Sciences, North Carolina State University, Raleigh, NC, United States; ^8^ Department of Nursing and Midwifery, Faculty of Medicine, Masaryk University, Brno, Czechia

**Keywords:** male infertility, spermatogenesis, steroidogenesis, endocrine disrupting chemicals, bisphenol, spermatozoa, perfluoroalkyl substances

## Abstract

Male fertility has been deteriorating worldwide for considerable time, with the greatest deterioration recorded mainly in the United States, Europe countries, and Australia. That is, especially in countries where an abundance of chemicals called endocrine disruptors has repeatedly been reported, both in the environment and in human matrices. Human exposure to persistent and non-persistent chemicals is ubiquitous and associated with endocrine-disrupting effects. This group of endocrine disrupting chemicals (EDC) can act as agonists or antagonists of hormone receptors and can thus significantly affect a number of physiological processes. It can even negatively affect human reproduction with an impact on the development of gonads and gametogenesis, fertilization, and the subsequent development of embryos. The negative effects of endocrine disruptors on sperm gametogenesis and male fertility in general have been investigated and repeatedly demonstrated in experimental and epidemiological studies. Male reproduction is affected by endocrine disruptors via their effect on testicular development, impact on estrogen and androgen receptors, potential epigenetic effect, production of reactive oxygen species or direct effect on spermatozoa and other cells of testicular tissue. Emerging scientific evidence suggests that the increasing incidence of male infertility is associated with the exposure to persistent and non-persistent endocrine-disrupting chemicals such as bisphenols and perfluoroalkyl chemicals (PFAS). These chemicals may impact men’s fertility through various mechanisms. This study provides an overview of the mechanisms of action common to persistent (PFAS) and nonpersistent (bisphenols) EDC on male fertility.

## Introduction

Male fertility has been declining over time. The temporal trend analysis revealed significant decline of more than 50% in total sperm counts between 1973 and 2011 (Levine et al., 2017). In this study, the assessment of male fertility was largely limited to western countries such as United States, Australia, and Europe. Study that is more recent focused on Asian men during last 50 years (1965–2015) and observed 20% decline in sperm concentration (Sengupta et al., 2018). That is, especially in countries where an abundance of chemicals called endocrine disrupting chemicals (EDC) has repeatedly been reported, both in the environment and in human matrices. Humans are unavoidably exposed to variety of chemicals having the potential to disrupt endocrine homeostasis. Particularly male reproductive system is susceptible to environmental stressors including persistent and non-persistent EDC such as PFAS and bisphenols. In several studies, the continued decline of semen parameters was correlated with immediate and long-term exposure to several EDC (Nordkap et al., 2012; Levine et al., 2017; Rehman et al., 2018).

EDC can be found in a variety of everyday products and goods. The widespread distribution of EDC causes that humans are continuously exposed through multiple sources including diet and drinking water. PFAS represents broad class of fluorinated chemicals with significant health concerns due to their diversity and persistence in the environment and in humans with elimination half-lives of several years (Olsen et al., 2007). From recent review articles, it is apparent that people worldwide are exposed to several PFAS (De Felip et al., 2015; Wang et al., 2018; Calafat et al., 2019). A main source of PFAS to humans and the environment is their production and use in industrial and professional applications. The major route of exposure to humans is the release of PFAS from consumer products, such as textiles, polishing and cleaning products, cosmetics, and food contact materials. The magnitude of exposure to PFAS differs across continents and even at the country level. The most frequently detected PFAS in human samples worldwide is PFOS (perfluorooctane sulfonate) and PFOA (perfluorooctanoic acid). For example, in the serum samples of German population from 2019, the concentrations of PFOA and PFOS ranged between 2 and 6 × 10^−9^ M and 2–10 × 10^−9^ M, respectively (Göckener et al., 2020). In China, median serum concentrations of PFOA (36 10 × 10^−9^ M) and PFOS (30 10 × 10^−9^ M) were significantly higher (Duan et al., 2020). The published scientific literature supports associations between PFAS exposure and adverse reproductive outcomes even at 10^−9^ M concentrations (Joensen et al., 2009; Di Nisio et al., 2019; Pan et al., 2019).

Bisphenols are associated with polycarbonate plastics, food packaging, food cans, thermal receipts, glass frames electronic equipment and many others. The level of human exposure to bisphenols and particularly to bisphenol A (BPA) has been investigated in several human biomonitoring studies all over the world. The measured urinary BPA in adult populations worldwide show very similar levels. For example, creatinine-adjusted median urinary levels of BPA in the adult U.S. population (*N* = 1808) from the National Health and Nutrition Examination Survey (NHANES) 2013–2014 were 1.20x10^−8^ M (Lehmler et al., 2018). Levels of 2.45 x10^−8^ M (*N* = 1400) were reported in France 2014–2016 (Fillol et al., 2021). In samples collected in 2010 in several Asian countries BPA urinary concentration ranged from 2.45 to 0.58 × 10^−8^ M (Zhang et al., 2011). Several studies have shown that even low exposure levels of BPA can pose adverse effects on male fertility (Zhang et al., 2011).

**FIGURE 1:**
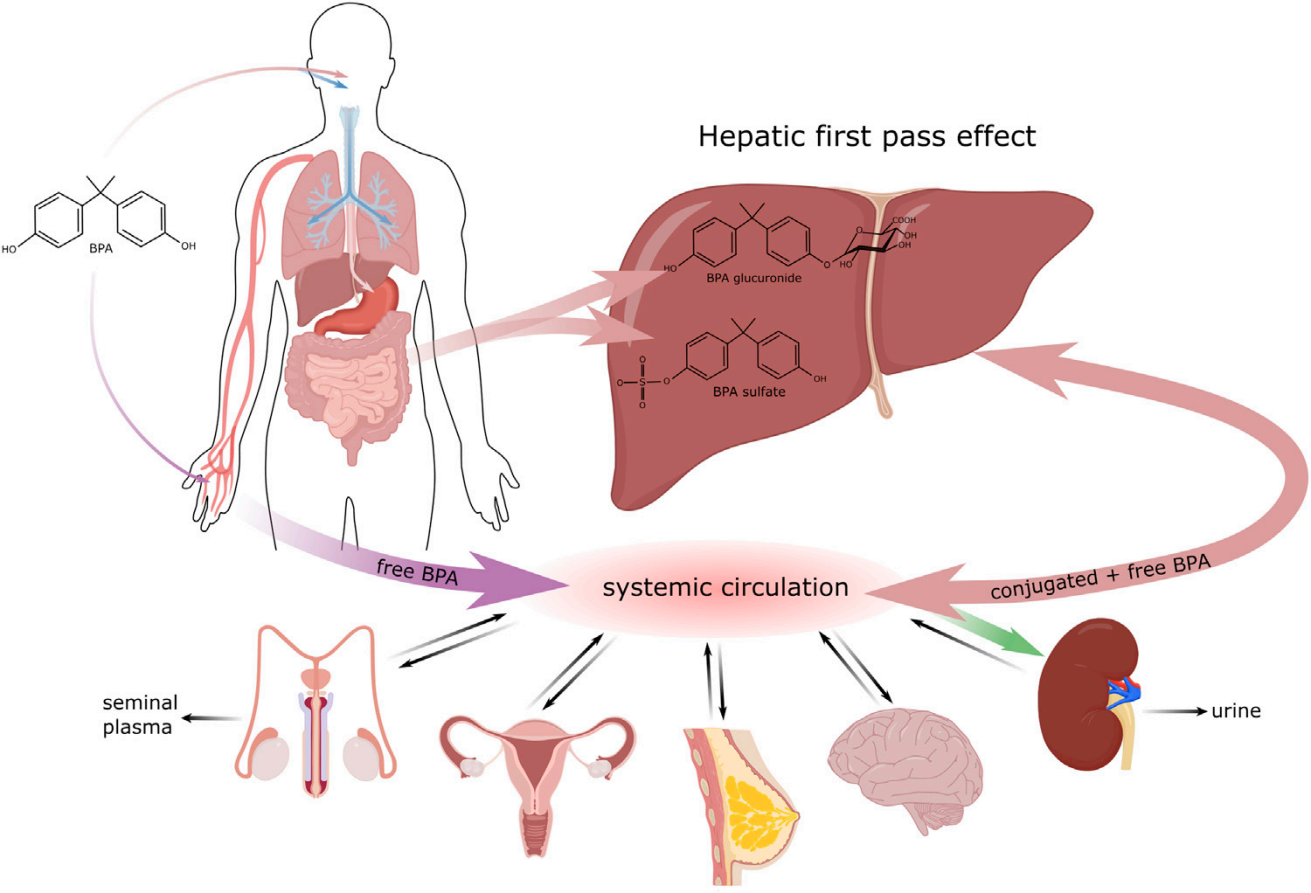
Biotransformation of BPA in the organism.

About a thousand chemicals are suspected to interfere with endocrine functions. Regarding male infertility, of particular interest are bisphenols, phthalates, and perfluoroalkyl and polyfluoroalkyl substances (PFAS) (Joensen et al., 2009; Li et al., 2011; Cai et al., 2015; Radke et al., 2018; Ješeta et al., 2019a). Therefore, the objective of this study is to provide an overview of the literature concerning the effects and mechanism of action of most abundant persistent (PFOS, PFOA) and non-persistent (bisphenol A) EDC on the male reproductive system (Figure 1) including: 1) effects on estrogen receptors, 2) anti-androgenic effects, 3) epigenetic effects on spermatogenesis, 4) effects of reactive oxygen species (ROS) production on sperm cells, 5) direct effects on sperm cells, and 6) effects on the blood-testis barrier. In addition, we outline the main entry routes of EDC into the organism.

## Exposure Routes of Endocrine Disruptors

There are three major routes, by which EDC can enter the organism. The most common pathway is per os; i.e., through the consumption of food and drinks (Geens et al., 2012) that were stored in containers or bottles made of plastic materials containing for example bisphenol A (BPA) in the form of polymer. Mainly after exposure to high temperatures, its monomers can be released into the contained food or drink and be ingested into the body (Le et al., 2008). In addition, for example dental fillings can contribute to the oral administration of BPA though minimally, by releasing this polymer into saliva shortly after the filling is applied (Joskow et al., 2006). Since BPA is a compound that has been well characterized in this context, we will use it as an exemplar with respect to the biotransformation of EDC in the organism. BPA biotransformation is shown in Figure 1.

After ingestion by the organism, most BPA is metabolized quickly in two possible ways, glucuronidation and sulfonation (Thayer et al., 2015). The greatest amount of BPA is metabolized in the liver, most frequently by the enzyme UDP-glucuronosyltransferase2B15 (UGT2B15), to its biologically inactive form BPA-glucuronide (Hanioka et al., 2008). This form is water soluble and is excreted in urine with a 5.4–6.4 h elimination half-life (Castellini et al., 2020). A portion of the received bisphenol is metabolized by sulfotransferase to BPA-sulphate. To a lesser extent, glucuronidation can take place outside the liver, in the intestine and partially also in the kidneys (Lušin et al., 2012). The remaining bisphenol A that was not metabolized in the gastrointestinal tract enters the bloodstream, where it is present in its free, bioactive form. The bisphenol ingested per os goes through a relatively effective detoxification process in the gastrointestinal tract 99% of it being eliminated within 24 h (Thayer et al., 2015). Studies by Guignard et al. (2016) and Gayrard et al. (2013) also point to the possible absorption of bisphenol in the oral cavity, thereby evading the primary metabolic processing in the liver. Nevertheless, Teeguarden et al. (2015) state that the concentration of free BPA in the bloodstream increases only negligibly after sublingual absorption.

Entry through skin has been studied intensely and discussed mainly in association with thermal paper used in the form of receipts (Thayer et al., 2016). Unlike in plastics, where bisphenol is present mainly in its polymerized form, thermal paper contains BPA in its free form in milligrams per gram of paper (Biedermann et al., 2010). Percutaneous entry is fast and is even accelerated by antibacterial gels that disrupt the dermal barrier. Bisphenol absorbed through skin evades primary metabolization in the liver and this significantly increases the concentration of free, bioactive forms of bisphenol in the bloodstream (Hormann et al., 2014). In the general population, approximately 1 μg of BPA is transferred per 1 finger after holding thermal paper for 5 s, with wet or greasy fingers increasing it to 23 μg per finger (Biedermann et al., 2010). In the study of Sasso et al. (2020), the half-life of free d6-BPA after dermal contact was determined to be up to 17.6 h, which is three times longer than after oral administration. Bisphenols that enter the body after dermal contact are excreted more slowly. Liu and Martin (2017) reported that labelled BPA was still detectable in urine 51 h after dermal contact.

Another possible route of entry into the body is inhalation. Most exposed are workers in factories producing goods containing BPA (Hines et al., 2018), while for most of the population this pathway is less important that the dermal or per oral routes (Geens et al., 2009).

The adverse effects of endocrine disruptors are often described in association with prenatal and perinatal exposition (Caserta et al., 2008). Many EDC are capable of placental transport and so free, bioactive forms of bisphenol from the mother’s blood can enter the bloodstream of the developing fetus. Furthermore, BPA-glucuronide is deconjugated to the free BPA form in the placenta (Nishikawa et al., 2010). Unlike in adult humans, the expression of UGT (enzymes responsible for BPA glucuronidation) in fetuses is minimal (Gerona et al., 2013). The transfer of free bisphenol from the bloodstream to the breast of a breastfeeding mother also poses a risk. High concentrations (10^–9^ M) of free bisphenol were detected in maternal milk a few hours after oral intake (Dualde et al., 2019).

Bisphenols that are not metabolized in the small intestine or primarily in the liver enter the circulation in their unbound form. Hovewer their lipophilic character allows their distribution to various targets, such as adipose tissue (Vénisse et al., 2019), the brain (Van der Meer et al., 2017), breast tissue (Dualde et al., 2019), the reproductive organs (Karrer et al., 2018; Ješeta et al., 2019b) or the prostate (Prins et al., 2014). During their detoxication bisphenols are conjugated with glucuronide or sulfate. Glucuronidation is a major elimination process that converts bisphenols to hydrophilic molecules that are excreted in the urine (Karrer et al., 2018). Many times, these substances have been detected in seminal plasma in concentrations ranging from 10^–8^–10^–10^ M (Hampl et al., 2013; Vitku et al., 2016). Seminal plasma is mainly the product of the accessory glands: seminal vesicles (65–75%), the prostate and epididymis (20–30%), and the bulbourethral and periurethral glands (1%) (Samanta et al., 2018). Therefore, it can be assumed that the primary way bisphenols enter seminal plasma is by means of blood transport to the accessory glands and their subsequent secretion. Transport through the blood-testis barrier (BTB) makes only a minor contribution to the total concentration of bisphenols detected in seminal plasma (Cheng et al., 2011).

Similarly, to bisphenols human exposure to PFAS occurs through ingestion, inhalation, and dermal contact (Sunderland et al., 2019). In contrast to bisphenols PFAS are bioaccumulative, and biopersistent, with long half-lives in humans (e.g., 3.5 and 4.8 years for PFOA and PFOS, respectively) (Olsen et al., 2007).

## Main Effect of Endocrine Disruptors on Male Fertility

**FIGURE 2:**
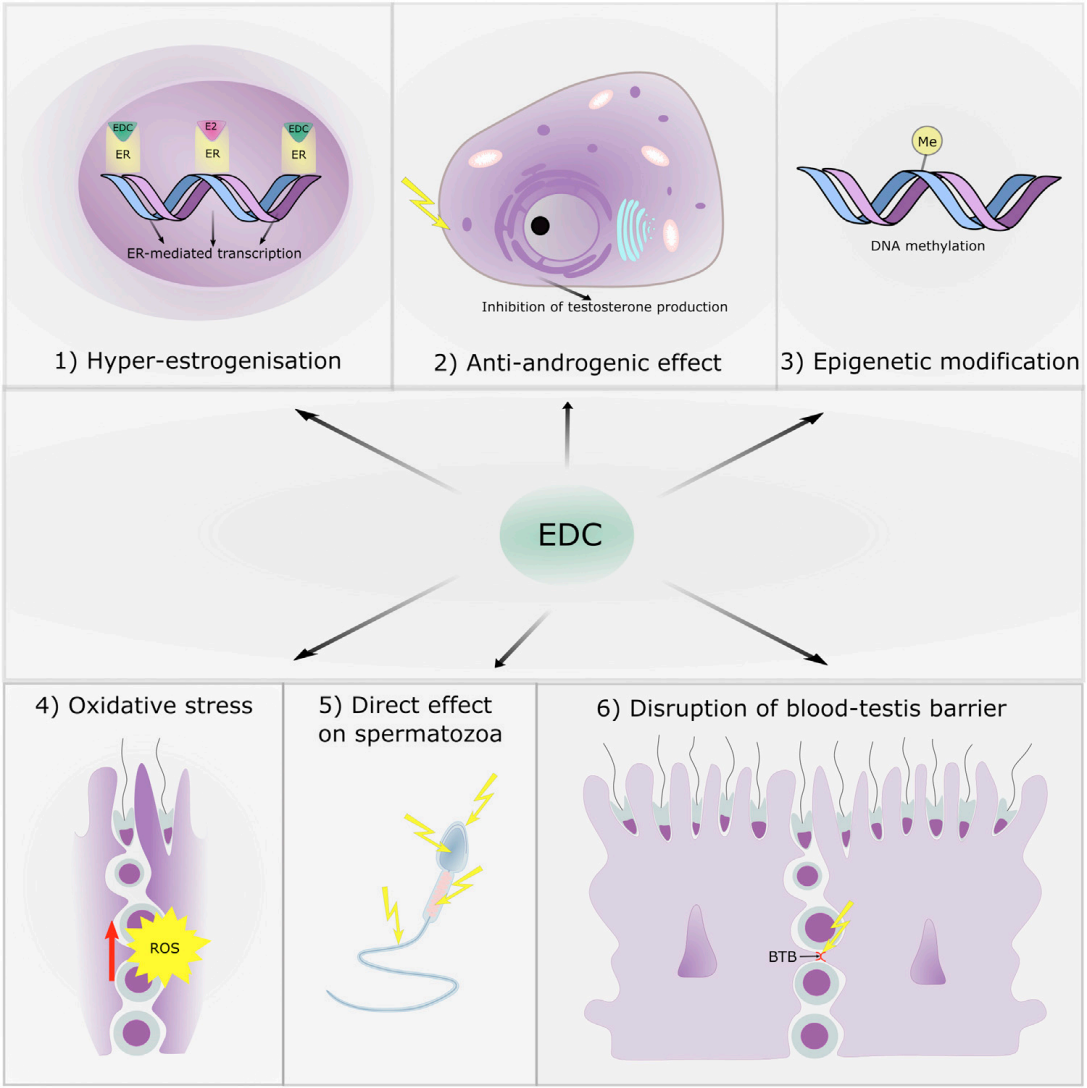
The main targets of endocrine disruptive chemicals with respect to male reproduction.

In this part, we summarize 6 main kind of effects: 1) effects on estrogen receptors, 2) anti-androgenic effects, 3) epigenetic effects on spermatogenesis, 4) effects as producer of reactive oxygen species (ROS), 5) direct effects on sperm cells and 6) effects on the blood-testis barrier. It is likely, that these effects intermingle and that clinical manifestations are the results of their various combinations. The main targets of EDC with respect to male reproduction are shown in Figure 2.

### Impact on Estrogen Receptors: Hyperestrogenization

Estrogen and androgen receptors are major targets of EDC. Bisphenols are small molecules, which, in competition with estrogen E2 and beta estradiol, affect many physiological functions via estrogen receptors. This leads to the disruption of steroidogenesis (Lagarde et al., 2015). BPA may affect Leydig cells via estrogen receptors (ESR1 and 2) and estrogen—related receptors (ERRα, β, γ), but probably mainly via the G protein-membrane estrogen receptor, all-of-these expressed in Leydig cells (Li et al., 2020). G-protein coupled estrogen receptor is a main target of the estrogen-like effect of BPA (Ge et al., 2014; Fitzgerald et al., 2015), as well as the alternative bisphenols (Cao et al., 2017). The estrogen-like and estrogenic effects of BPA have been described in germ cells (10^–5^ M, Karmakar et al., 2017), ovarian cells (10^–10^ M, Shi et al., 2017) and testicular cells (10^–5^ M, Wang H. et al., 2017; 10^–9^ M, Ge et al., 2014). The chemical structure of bisphenols is very similar to that of estradiol; it causes estrogenic activities resulting in disruptive effects on the feedback regulation of the hypothalamic–pituitary–gonadal axis. This leads to the decreased secretion of luteinizing hormone (LH) in the pituitary gland, and finally, the hypostimulation of Leydig cell steroidogenesis results in the lower production of testosterone, which plays a crucial role in fetal development, sexual functions, and spermatogenesis.

In addition, some persistent PFAS, such as PFOS and PFOA also exhibit certain endocrine disruptive activity. For instance, weak estrogenic activity was observed in animal and *in vitro* studies (Benninghoff et al., 2011; Sonthithai et al., 2016). As observed *in vivo* PFOS and PFOA might exert their estrogenic effects via the activation of estrogen receptors (10^–5^ M, Kjeldsen and Bonefeld- Jørgensen, 2013). Recently, *in vitro* studies have shown that PFOS might have the ability to interact with ERα and enhance ERα-dependent transcriptional activation (Benninghoff et al., 2011).

### Impact on Androgen Receptors: Antiandrogenic Effect

In addition to an effect on estrogen receptors, a direct antiandrogenic effect has also been detected. The presence and correct ratio of steroid hormones is determinative for the correct initiation of male gonad development and the masculinization of the sex ducts. Leydig cells (LC) produce two hormones (testosterone and INSL3).

Leydig cells synthetize testosterone from the steroid cholesterol by means of the activation of the PKA signaling pathway, this leading to the transport of cholesterol to mitochondria, where pregnenolone is formed and subsequently transported to the smooth endoplasmic reticulum for the synthesis of testosterone. In higher concentrations, BPA affects the production of testosterone directly by disrupting enzymes engaged in the endoplasmic reticulum. The endocrine disruptor BPA (10^–6^ M) directly inhibits the enzymatic activity of CYP17A1, HSD3B and HSD17B3 engaged in the production of testosterone in rats and humans (Ye et al., 2011). A study on the effect of BPA (10^–5^ M) on the GW7-12 *in vitro* line (human fetal testicular cells) revealed a significantly reduced production of testosterone (Maamar et al., 2015). The exposure of Leydig cells to BPA (10^–11^ M) was shown to block the production of testosterone, androgen biosynthesis and CYP17 gene expression in rats (Akingbemi et al., 2004). BPA (10^–6^ M) also reduced the mRNA level of fetal Leydig cell-related genes (Star-steroidogenic acute regulatory protein, 3 beta hydroxysteroid dehydrogenase/Delta, cytochrome 540 family 17 subfamily A member 1 (Cyp17a1), INSL3) in mouse testicular explants cultivated for 3 days (Eladak et al., 2015). In addition, MEHT (a reactive phthalate metabolite) has been shown to activate the PPAR (peroxizome-prolifeartion activated receptor) signaling pathway and via activation of the PPAR: RXR receptor, also to inhibit the transcription of aromatase, an enzyme important for the development of the reproductive system (Men et al., 2019; Xie et al., 2019). Moreover, by means of PPAR activation, MEHT impaired thetranscription of the steroidogenic genes StAR and p450c17. StAR is a mediator of cholesterol transfer to the mitochondria of Leydig cells and p450c17 is a steroid-converting enzyme. Dysfunction in these proteins negatively influenced testosterone production (Rahman et al., 2017).

Testosterone is metabolically activated to dihydrotestosterone (DHT) by 5α-reductase, which is essential for the development of the male genital tract. EDC often act as inhibitors of 5α-reductase, which are necessary for conversion of androgens (Sweeney et al., 2015). Indeed, BPA inhibits the production of testosterone in fetal testicles. In gravid mice exposed to BPA, a similar effect was observed for estrogen, thus downregulation of FLC genes such as Scarb, Star, Cyp11a1, Cyp17a1, and Svs5. A negative impact on the weight of testicles was also described (Naciff et al., 2005). It is well known that the disruption of testosterone production leads to abnormalities such as hypospadias or a small penis (Brouwers et al., 2006; Conlon, 2017).

Further, an adverse effect of PFAS on the fertility of male mice was reported. The first study reporting on PFOS-induced infertility in mice elucidated multiple effects including effects on sperm counts, testosterone serum concentration, and the expression of gonadotropin gene receptors. In this study mice were administrated daily doses of 0, 2, 10, or 20 mM PFOS for 7, 14, or 21 days. After 21 days of exposure to the highest dose, the study recorded a significant decrease in sperm counts, in the expression levels of gonadotropin receptors and in testosterone serum concentration (Wan et al., 2011). The findings were supported by several other studies conducted in mice and rats. For example, a significant dose-dependent decrease in sperm counts was observed after 28 days of exposure to PFOS concentrations ranging from 5 to 100 mM (Qiu et al., 2013). Decreased serum testosterone levels were also observed in rats after 28 days of exposure to PFOS at a daily dose of 10^−3^–10^−2^ M after 28 days (López-Doval et al., 2014). Another study reported testicular toxicity induced by PFOS exposure that manifested in a significant decrease in serum testosterone concentration and sperm counts after 5 weeks exposure at 20 mM/day. Moreover, increased levels of PFOS (1-20 × 10^−3^ M) led to alterations in the expressions of testicular estrogen receptors (Qu et al., 2016). Thais is true for other perfluoroalkylated substances; for instance, PFOA also caused a decline in serum and testicular testosterone levels and an increase in serum estradiol levels in adult male rats after 2 weeks of exposure (Cook et al., 1992; Biegel et al., 1995). The decline in testosterone production in rats can be explained by the inhibition of testicular microsomal steroidogenic enzymes in Leydig cells, specifically by the inhibition of 17β-hydroxysteroid dehydrogenase and 3β-hydroxysteroid dehydrogenase (Zhao et al., 2010). Adverse effects were observed even for *in utero* exposure. For example, exposure to doses of 1 × 10^−4^ M and 4 × 10^−4^ M of PFOS during the prenatal period caused a significant drop in testosterone concentration and the number of fetal Leydig cells in rats (Zhao et al., 2014). Prenatal exposure to PFOA concentration ≥24 × 10^−3^ M affected sexual maturation in male mice (Lau et al., 2006).

The second hormone produced by Leydig cells is INSL3 (insulin-like 3), which is released into the circulation and is crucial for testicular descent, which is disrupted by INSL3 knockdown (Zimmermann et al., 1999). Some EDC such as xenoestrogen diethylstilbestrol (DES) impair the production of this hormone and cause cryptorchidism. This was observed from the 1940s–to the 1970s, when xenoestrogen diethylstilbestrol (DES) given to pregnant mothers led to a high incidence of cryptorchidism in male children (Palmer et al., 2009). Similarly, in an experimental study, DES given to pregnant mice caused bilateral cryptorchidism and the downregulation of Insl3 gene expression in fetal testes (Emmen et al., 2000).

Bisphenols can also exert direct harmful effects at the testicular level. In rat Sertoli cells, BPA caused the activation and upregulation of Raf1 and *p*-ERK1/2, probably through the steroid receptors. It can be assumed, that the effect of BPA on Sertoli cells is via the estrogen-ERK signaling pathway (Li et al., 2009). Indeed, the similar phthalate DBP decreases the expression of SCF and the c-kit receptor, which are very important for the correct interaction between Sertoli cells and gonocytes. Such an effect leads to the disruption of Sertoli cell proliferation during the prenatal period (Žalmanová et al., 2016). Fiorini et al. (2004) also reported that BPA affected intercellular junctions in the Sertoli cell line SerW3.

### Epigenetic Modifications

EDC are often present in very low doses without any clinical manifestations. Such a situation can lead to crypto effects such as “idiopathic” infertility or other health problems with non-specific symptoms. Post-translational modifications of some crucial proteins, particularly the regulating epigenetic factors, seem to be a common action of these very low doses. Many pathways of EDC action result in epigenetic changes to DNA. Although the sequence of nucleotides remains unaffected during exposure to EDC, changes in genome-wide methylation status, as well as the silencing or enhancing of individual genomic sites often follow such exposure. These epimutations result in the changed transcriptional activity of the genome, giving rise to many possible negative impacts, such as the failure to scavenge ROS and to repair DNA damage, as well as the disruption of mitochondrial biogenesis.

The mechanisms of EDC can be divided according to two basic effects: The first is the global effect, when EDC affect the occurrence of epigenetic regulators and their cofactors. The most studied mechanism is the effect of EDC on the levels of enzymes that regulate DNA methylation, especially the levels of DNA methyl transferase (DNMT). The probable mechanism of the effect of EDC on DNMT is the direct regulation of DNMT mRNA expression by the target receptors affected by EDC.s The second is the gene-specific effect, when EDC modulate the regulation of locus-specific epigenetic modifications. This is a very frequent EDC induced epigenetic change. The gene- and locus-specific effects are unclear and largely unknown, but one probable route is via interference with a nuclear receptor function. Locus-specific chromatin states have been shown to be regulated by nuclear receptors (of estrogen or androgen) (Martens et al., 2011). Nuclear receptors such as estrogen receptors, the retinoic acid receptor, and Ah receptors can regulate DNA methylation patterns by interacting with DNMT (Kouzmenko et al., 2010) or thymine DNA glycosylase (Hassan et al., 2017). The impact of Bisphenol A is the most studied. It has been shown that bisphenol A (10^−8^ M) affects DNA methylation of the Fkbp5 gene in mice in an ERβ-dependent manner (Kitraki et al., 2015). Furthermore, the changes in DNA methylation observed in the reproductive system of adult female mice exposed prenatally to Bisphenol A showed a 93% correlation with ERα binding sites (Jorgensen et al., 2016).

Of particular note is epigenetic transmission to further generations. This phenomenon is induced by many modifications, such as DNA and histone methylation, histone acetylation and other post-translational modifications of core histones, as well as by epigenetic writers and erasures, and translational factors, for example. The molecular impact of individual EDC is still unknown.

The exposure of somatic cells to EDC can cause health problems for the exposed people, but gamete exposure to EDC can lead to intergenerational effects where the problem is transduced to subsequent generations of offspring (Pietryk et al., 2018). In addition, intergenerational and transgenerational inheritance occurs due to a change in the epigenetic code of germ cells. For example, in the period from 1940s–to the 1970s, synthetic estrogen diethylstilbestrol (DES) was used in women during early pregnancy to prevent miscarriages, however this treatment increased the risk of a rare form of vaginal clear cell adenocarcinoma and infertility in daughters (DES daughters) (Conlon, 2017). Interestingly, an increased risk of hypospadias, an abnormality of the male reproductive system, was also found in sons of DES daughters (Brouwers et al., 2006). Indeed, exposure to bisphenols impairs DNA methylation and the histone code in oocytes (Wang et al., 2016; Nevoral et al., 2018). DNA methylation is also potentially affected by environmental pollutants in spermatozoa (Lu et al., 2018), which may lead to aberrant gene imprinting (Zhang et al., 2012; Doshi et al., 2013). In addition, it was found that estrogen-like EDC have a negative effect on sperm probably by means of a direct impact on the sperm histone code. Similarly to estrogen and estrogen receptors, estrogen-like EDC are involved in the histone code establishment (Dumasia et al., 2017).

### Effect of Endocrine Disrupting Chemicals as Inducers of Reactive Oxygen Species Production on Spermatozoa

Ullah et al. (2018) showed that BPA and its analogues induce oxidative stress in testicular tissue of rats and therefore impair their reproductive functions. Exposure to BPA, BPB, BPF and BPS verifiably increases the activity of ROS and lipid peroxidation (LPO), which leads to oxidative stress *in vitro* and significant DNA damage in rat spermatozoa *in vivo*. Increased LPO activity causes a loss of membrane integrity leading to increased cell permeability, the inactivation of enzymes and structural damage to DNA (Domínguez-Rebolledo et al., 2010). Among other effects, the production of ROS can activate apoptosis directly, triggering the enzymatic cleavage of DNA (Muratori et al., 2015). In samples of spermatozoa incubated with these EDC, high activity by superoxide dismutase was observed, this antioxidant enzyme probably utilized for the neutralization of free radicals (Ullah et al., 2019). The induction of oxidative stress by BPA and BPS in sperm cells and reproductive tissues and the subsequent increased activity of superoxide dismutase was also documented in other studies (10^−4^ M, Barbonetti et al., 2016; 10^−4^ M, Wang et al., 2016). The *in vitro* exposure of mouse Sertoli cells to BPA induced the excessive production of ROS and cellular oxidative stress. The overproduction of ROS in cytoplasm resulted in mitochondrial dysfunction contributing to the alteration of mitochondrial permeability and the apoptosis of Sertoli cells (10^–5^ M, Wang C. et al., 2017).

The BPA-induced production of ROS is probably one of the mechanisms of steroidogenesis disruption in Leydig cells (D’Cruz et al., 2012). Similarly, negative effects of PFOS on male mice fertility have been detected. In addition, oxidative stress reported after exposure to PFAS may be involved in the disturbance of BTB and in male reproductive injury. Both PFOA and PFOS can trigger the expressions of nuclear hormone receptors particularly peroxisome proliferator-activated receptor PPARα and γ, and cause oxidative damage (10^−5^ M, Olufsen et al., 2014; Elcombe et al., 2012). Further exposure to PFOS increased p38 MAPK and decreased the BTB-related junction proteins Connexin43 and Occludin in mice testes (Qiu et al., 2013; Qu et al., 2016).

### Direct Effect on Spermatozoa

#### Endocrine Disrupting Chemicals and the Sperm Calcium Pathway and Motility

The action of EDC action on human CatSper can affect fertilization in several ways. EDC can evoke motility and acrosome reaction too early and in the wrong place (Schiffer et al., 2014). In addition, these substances can desensitize sperm to female factors that normally activate sperm. The motility and viability of sperm, along with their mitochondrial functions, and intracellular ATP levels, are negatively affected by BPA through the activation of MAPK, phosphatidylinositol 3-kinase and, protein kinase A (10^−4^ M, Rahman et al., 2016). Several studies demonstrated that some EDC are able to activate the sperm-specific CatSper channel and induce calcium ion influx, which modulates functional sperm parameters (10^−10^ M, Zou et al., 2017; Tavares et al., 2013).

It was shown that 4-Methylbenzylidene camphor (4-MBC), 3-benzylidene camphor (3-BC), di-*n*-butyl phthalate (DnBP), nonylparaben (n-NP), α-zearalenol, padimate O (OD-PABA), benzophenone-3 (BP-3), homosalate (HMS), and 4,4′-DDT all release Ca ions via the CatSper channel (Schiffer et al., 2014). Indeed, bisphenols (BPG, BPAF, BPC and BPB) are also able to induce Ca^2+^ signals via the CatSper channel and also inhibit progesterone induced Ca^2+^ signals in human sperm cells. CatSper is normally activated by progesterone produced by cumular cells (Rehfeld et al., 2020). CatSper is also activated by p,p′-DDE, a metabolite of dichlorodiphenyltrichloroethane (DDT) (Tavares et al., 2013). This activation is important for sperm penetration and men lacking functional CatSper are sterile (Brown et al., 2018). BPA at concentrations of 10^−9^–10^−6^ M could affect human sperm motility parameters, while BPA at a concentration of 10^−6^ M could induce a rapid, though transient increase in [Ca^2+^]i in human sperm cells (Kotwicka et al., 2016). It was observed that 4-MBC similarly to progesterone induces spermatozoa hyperactivation and that other EDC (3-BC, TCS a 4-MBC) induce acrosomal exocytosis (Schiffer et al., 2014). As demonstrated by an *in vitro* study using mature human sperm, PFOA exposure (2.5–25 μg/ml) activated the CatSper channel and triggered a transient rise in human sperm [Ca^2+^]i (Yuan et al., 2020).

It is well known that high gonadotropin concentrations are associated with disruption of spermatogenesis. Indeed, the disrupting mechanism of BPA consists of the alteration of several signaling pathways and leads to changes in protein kinase A activity, protein tyrosine phosphorylation, ATP generation, and the activities of oxidative stress-related enzymes (succinate dehydrogenase, peroxiredoxin-5, glutathione peroxidase 4). These enzymatic activities are determinative for sperm motility and penetration and the overall ability of sperm to fertilize an oocyte (Rahman et al., 2015; Rahman et al., 2017; Rahman et al., 2018).

#### Endocrine Disrupting Chemicals and Sperm Integrity

A recent study of recruited subfertile patients and matched controls showed, that urinary BPA concentrations were associated with declined semen quality and increased sperm DNA damage, especially in patients with multiple defects, as well as with increased oxidative stress indicators (10^–4^ M Omran et al., 2018).

Zatecka et al. (2014) observed that the exposure of outbred mice to TBBPA (tetrabrombisphenol A) during gestation led to a significant reduction in T4 and T3 levels and the ratios of protamine 1 to protamine 2, an increase in the total protamine/DNA ratio in spermatozoa, and a higher proportion of apoptotic spermatozoa. According to the DNA protection hypothesis for protamines, the prediction would be that the any detected increase in DNA damage would correlate with decreased total protamine 1 + protamine 2/DNA ratios (Aoki et al., 2005; Oliva, 2006). Ullah et al. (2018) showed that BPA and its analogues caused oxidative stress in testicular tissue and disrupted reproductive functions in rats. It has been documented that exposure to all these bisphenols (BPA, BPB, BPF and BPS) increased the activity of ROS leading to oxidative stress *in vitro* and significant DNA damage in spermatozoa. Bisphenols also increased LPO activity, which can lead to increased cell permeability, and fragmentation of the DNA structure (Domínguez-Rebolledo et al., 2010). Indeed, o-quinone, a reactive metabolite of BPA, can bind DNA by means of covalent bonds and, in the presence of a peroxidase activation system, it can also produces toxic adducts (Atkinson and Roy, 1995).

However, a hospital-based study has shown conflicting results with respect to measurements of BPA in urine, which mainly contained conjugated BPA, and correlations with semen quality and sperm DNA integrity (Vitku et al., 2016). In a US study of young men recruited from the general population, no associations were found between urinary BPA contents and spermiogram parameters, apart from a strange negative association between BPA and sperm DNA fragmentation (Goldstone et al., 2015). In another clinical study, a relationship was found between BPA level in urine and semen-quality parameters in men with at least one parameter below the WHO reference levels when compared to men with normozoospermia (i.e., with all parameters above the WHO reference levels) (10^–5^ M, Meeker et al., 2010). In a study conducted among young men (median age, 19 years) (*n* = 105), high serum concentrations of PFAS were significantly associated with reduced numbers of normal spermatozoa (Joensen et al., 2009 (PFOS 24,5 ng/ml; PFOA 4,9 ng/ml; PFHxS 6,6 ng/m,l)). However, sperm parameters such as concentration, total sperm count, and sperm motility showed statistically insignificant downward trend in men with high PFAS levels. This study also reported a trend toward lower inhibin B/FSH ratio with high PFAS levels. Another cross-sectional study of 247 men reported a negative association between serum PFOS and testosterone levels (Joensen et al., 2013 (8,46 ± 3,74 ng/ml PFOS)). A study conducted in China among a population of adult men (*n* = 664) collected matched semen and serum samples to examine multiple semen parameters along with 16 target PFASs measured in semen and serum. However, the associations observed between levels of PFAS in serum and semen parameters were statistically weak, except for DNA stainability, which was more strongly related to serum PFAS than to semen PFAS (Pan et al., 2019).

### Disruption of the Blood-Testis Barrier

The integrity of the BTB in the testicles is very important for the proper development of spermatozoa. The BTB is necessary for the division of the germinal epithelium into compartments the basal and adluminal. Spermatogenic germinal cells undergo mitosis and cross the BTB from the basal to the adluminal compartment to facilitate germ cell release (Lie et al., 2009).

In the seminiferous tubules, the neighbouring Sertoli cells form an integral part of the BTB. The very tight connections between the cells are represented mainly by tight intercellular junctions, adherens junctions, desmosomes and gap junctions. The key process relating to this barrier is when spermatocyte I at the proleptotene stage passes through it. This transfer is important for spermatocyte I to undergo structural changes. The barrier protects sensitive post-meiotic germ cells from exogenous toxicants from blood. Moreover, if this barrier did not tighten well or was damaged, antibodies to sperm cells would be produced, which could ultimately result in male infertility (Cheng and Mruk, 2012). Effects of EDC on the BTB barrier can directly negatively affect spermatogenesis in adults as well as the embryonic development of testicular tissue.

Disruption of the hematotesticular barrier by BPA has been reported. These substances disrupt the synthesis of proteins (occludin, clausin—11, N-cadherin, connexin 43) that are necessary for the presence of tight intercellular junctions between neighbouring Sertoli cells. This leads to dysfunction of the intercellular connections and the overall disruption of BTB integrity, which may cause development of an autoimmune disorder (10^–4^ M, Li et al., 2009).

It has been reported previously that BPA also affects anchoring junctions that attach spermatids to Sertoli cells (Toyama et al., 2004). This effect of BPA can result in the Sertoli cells only syndrome, when the adhesion or communication proteins between Sertoli cells and spermatogonia are downregulated. BPA impairs male reproductive health via the perturbation of Sertoli cell tight junctions, the downregulation of blood-testis barrier protein-activated ERK1/2 (i.e., activated by the proteins JAM-A, ZO-1, N-cadherin, and connexin 43) and the redistribution of intercellular connection proteins (Li et al., 2009; Gao et al., 2015). *In vitro* methods revealed that the mechanism of BPA action on Sertoli cells is realized via interferences with junctional proteins such as occludin, connexin 43 and E-cadherin (10^–5^ M, Fiorini et al., 2004). It has been reported previously that BPA affects microfilament distribution, F-actin organization, and the retraction of actin microfilaments in human Sertoli cells (10^–5^ M, Xiao et al., 2014). This was associated with the mislocalization of actin regulatory proteins, leading to the failure of Sertoli cell blood-testis barrier function. This microfilament effect is also associated with disruption of the phagocytic function of Sertoli cells.

In studies performed *in vitro*, BPA induced mitochondrial dysfunction10^−4^ M, apoptosis10^−5^ M, and DNA damage in Sertoli cells, along with the disruption of blood-testis barrier integrity (10^–4^ M, Adegoke et al., 2020).

In relation to PFAS, it has been confirmed that both PFOS and PFOA can cause damage to BTB integrity. The underlying molecular mechanism of PFOS induced BTB disruption is probably via the activation of the p38 MAPK/ATF2/MMP9 signaling pathway. Studies conducted *in vivo* and *in vitro* demonstrated that PFOS activated p38/ATF2 and disrupted the function of the Sertoli cell BTB (10^–5^ M**,** Qu et al., 2016; 10^–5^ M, Qiu et al., 2013). Likewise, PFOA activated p38 MAPK signaling pathway and primary Sertoli cells treated with PFOA showed changes in BTB-associated proteins (Lu et al., 2016).

Many of studies detected coctails of various EDC in human fluids from non-persistent to persistent in various concentrations. Nonpersistents EDC are ubiquitous chemicals containing in food and drink with regular daily intake on the contrary, non-persistent is typical of their accumulation in body fluids for several years. In context of their nonlinear and potentially synergistic effect it can lead to disruption of physiological sensitive processes like spermatogenesis or oogenesis. It was previously reported negative correlation between presence of EDC and sperm concentration in men (Zamkowska et al., 2018). Similarly it was observed negative effect of EDC on testicular cells during *in vitro* experiments (Akingbemi et al., 2004; Xiao et al., 2014; Wang H. et al., 2017) in similar concentrations like detected *in vivo* in human (Vitku et al., 2016).

## Conclusion

The negative impact of EDC on fertility has been demonstrated many times. However, it is important to distinguish between a model laboratory experiment and an *in vivo* situation. The effect of one substance or one group of substances is usually monitored in individual studies. In real life, however, many EDC of many different origins are commonly mixed many together. To date, more than 800 man-made chemicals have been discovered that are likely to interfere with the human endocrine system have been recognized. The question of mutual interactions between individual EDC is also very important. Most of these potential EDC have not been evaluated for their effect on human reproduction. Indeed, chemical companies release many new chemicals into the environment without careful verification of their effects on human reproduction. For example, rapid developments in chemical engineering have to led to the replacement of prohibited substances with new similar analogues suitable for industrial use. They are, however most frequently chemicals with unknown effects on human health or reproduction. Furthermore, these compounds can approved for use quite quickly—that is, any negative effects on health and reproduction are unambiguously proven., Thus, potentially damaging compounds are spread globally, leading to exposure to the general human population, including pregnant women and infants, Persistent and non-persistent EDC may affect the endocrine regulation of male reproductive axes. Understanding the mechanistic effects of EDC on male reproduction is crucial for detection and early implementation of preventive measures. In this work, we reviewed the major mechanisms of action common for both persistent and non-persistent EDC. Our results provided indication that both persistent and non-persistent EDC are associated with the formation of ROS, disruption of BTB, disturbance in testosterone production in Leydig cells, and interfere with estrogenic and antiandrogenic signaling at environmentally relevant concentrations (nanomolar).

In general, human exposure rarely occurs in isolation but rather to mixtures of EDC. The impact of the EDC mixtures may be insignificant, but it can also have a strong synergistic effect. Most of the EDC mixtures have not been evaluated for their effect on male reproduction and their combined mode of action is unknown. Therefore, future studies should examine the associations of mixtures of persistent and non-persistent chemicals with male fertility.
